# AI and business management: Tracking future research agenda through bibliometric network analysis

**DOI:** 10.1016/j.heliyon.2023.e23902

**Published:** 2023-12-29

**Authors:** Ashok Kumar Patra, Ashyashree Praharaj, Desul Sudarshan, Biswajit Prasad Chhatoi

**Affiliations:** a Department of Business Administration, Berhampur University, India; b Department of Library Science, Berhampur University, India

**Keywords:** Bibliometric analysis, Artificial intelligence, VOSviewer, R-Studio, Managerial decision making

## Abstract

This study has been designed to analyse the academic landscape of AI on the Scopus and Web of Science (WOS) indices and compare the findings. AI is one of the most prominent and preferred research areas, only a few studies are dedicated to the bibliometric aspect of it. There is a need to compare studies on AI over different databases to identify the impact and usefulness of those studies in decision-making in business management. To conduct this analysis, the authors have collected data from both Scopus and WOS. ‘VOSviewer’, ‘R-Studio’, and ‘MS Excel’ software have been used for performance analysis and science mapping. This is one of the exceptional studies which perform a comparative analysis between two indices and also identifies funding sponsors for support of research in AI. “*Dwivedi, Y.K.*” is the most productive author and “*Huang, Minghui*” is the most impactful author. “*National Natural Science Foundation of China*” is the funding agency which has significantly supported AI research. Technical aspects like “Machine learning”, “neural networks”, and “blockchain” with ‘Sustainability’, ‘sustainable development’, ‘accounting’, and ‘auditing’ are trending themes for managerial decision-making.

## 1 Introduction

Artificial Intelligence (AI) has been a part of business studies since the last decade, prioritising the use of smart technologies to obtain efficiency within organisations [1,2]. Pivotal aspects such as productivity, proactiveness, and effective decision-making are addressed by implementing AI in day-to-day business processes [3]. Several departmental functions such as sales and marketing [4,5], data analytics and decision support [6], human resource management [7], and sustainable development [8] rely on AI for effectual execution.

The practice of bibliometrics in research can be traced to 1917 [9]. The current trends in bibliometrics are event tourism [10], AI ethics and privacy [11], people analytics [12], knowledge network management [13], business intelligence and data analytics [14], and many more. Researchers across several domains have used bibliometrics as it enables studies with quantitative insights into a specific domain for a specified period [15].

This study addresses two prominent research gaps. Although AI is one of the most prominent and preferred research areas, only a few studies are dedicated to the bibliometric aspect of it. There is a need to compare studies on AI over different databases to identify the impact and usefulness of those studies in decision-making in business management. A negligible amount of study is found on performance analysis prioritising funding aspects of research.

The study has been designed to analyse the academic landscape of AI on the Scopus and Web of Science (WOS) databases and compare the findings. Performance analysis and science mapping have been used to recognise different networks and metrics. VOSviewer and R Studio have been used for the bibliometric workflow of 1256 Scopus, 209 WOS, and 1300 combined (Scopus & WOS) articles. The USA has produced the highest number of articles on AI. “*Technological Forecasting and Social Change*”, “*Journal of Business Research*” and “*Business* Horizon” are the prominent sources. “The *National Natural Science Foundation of China*” is the most prominent funding agency. “*Dwivedi, Y.K.*” and “*Huang, Minghui*” are the most productive and impactful authors. The study identified “machine learning”, “neural network”, “sustainable development”, and “blockchain” are emerging areas of research. The outcome of this study aims to benefit researchers, academicians, technology developers, decision-makers, and management enthusiasts in comprehending the academic landscape of AI.

A few distinct motivations fuel this study such as: (i) AI has been the subject of interest for the last few decades. It is quintessential to comprehend the scholarly publication in the said domain to evaluate the quality and impact of the articles. (ii) In present times, the widespread application of AI by several business organisations has engrossed our attention in evaluating the academic growth of the said domain with special reference to future implications. (iii) With the emergence of productivity tools such as ChatGPT, Bard by Google, etc., the researchers are motivated to analyse the parameters that developers keep into consideration to address organisational efficacy. Among the several techniques in the scientometrics discipline, the researchers have selected bibliometrics as their preferred tool to perform performance analysis, content analysis, and science mapping [16].

This study is the first to perform a comparative analysis of two herculean databases. This study also identifies the prominent funding agencies which sponsor and support research in the aforementioned area. The study's significance can be reflected through detailed performance analysis and comprehensive co-occurrence analysis to scrutinise the academic landscape of AI in business management.

This study has been tabulated into six salient sections. The opening section comprises an introduction to the study, followed by a detailed literature review. The methodology has been discussed in the next part, followed by data analysis and interpretation. The fifth section explains the research findings, followed by discussions, conclusions, limitations of the study, and future research courses.

## 2 Literature review

The concept of AI can be traced back to the early 1970s [17]. Apart from the traditional usage in the technology domain, AI also has implications in research and policy formulation [18], marketing [19], and customer engagement [20], tourism industry [21], supply chain management [22] and overall organisational development [23]. For the fintech industry, AI is pivotal in facilitating technology, such as blockchain, cryptocurrency, etc., into the traditional economic and legal environment [24].

The application of AI for business and allied management studies can be tracked in the recent decade. Studies by Refs. [[25], [26], [27]] highlighted the usage of AI in new product development, value creation, and sustainable economic development [28]. These studies have identified the positive impact of AI on the said areas [29]. identified a positive result in customer engagement for tech giants such as Amazon in the Indian context. However [30], highlighted the importance of entrepreneurial skills apart from AI to formulate effective strategies for sustenance in the competitive marketplace. Another study [31] foregrounded the increasing cases of cybercrimes, violation of privacy, growing economic injustice, and replacement of human labour as the negative impacts of AI. The authors believe that the positive implications are more compared to negative ones. Besides, AI's growing popularity and usage favour the positive impact more than the latter [32].

Innovative AI chatbot such as ChatGPT has been the “buzz” in recent times. A study by Ref. [33] accentuated the positive advancement of this bot in the changing job requirements, transforming the User Interface (UI) landscape and content development for social media platforms. Apart from the traditional utility, ChatGPT is an efficient tool for management research [34] and for supporting entrepreneurial ventures [35]. AI tools such as ChatGPT have been criticised for violating ethical conduct, especially in education and research, as they generate false text for academic purposes [36]. Apart from the fewer limitations, we believe that ChatGPT can be used to assist researchers in the respective studies across every domain.

Amongst the several criticisms in the domain of AI concerning employment generation, the study by [37] has identified the lack of awareness between the AI utility and the tech-savvyness of labourers. Awareness of the public concerning the four intelligence processes such as mechanical, analytical, empathetic, and intuitive is highly essential in eliminating the negative stigma of AI hampering employment opportunities [38]. The researchers believe there exists a dormant need for public awareness which provides transparent knowledge on the pros of AI and eradicates ignorance and negative stigma.

This study is channeled by a few limitations. Even though AI has been the most prominent topic of study, only a limited amount of literature is dedicated to the bibliometric approach that too with very few documents. The concept of AI has been introduced in the last five decades. Still, a negligible number of studies have been conducted on the performance analysis of the said area. A significant number of research has been performed across several research databases, but there is a need to compare the different parameters across databases. A latent need to identify the prominent funding agencies sponsoring research works in the field of business and allied management studies on AI.

## 3 Methodology

The present study is centered on “Artificial Intelligence” in academics. Data were collected from the Scopus [39] and ‘Web of Science (WOS)’ databases on May 27, 2023 using “Artificial Intelligence” as a keyword. Scopus and WOS stand out as the foremost provider of bibliometric data and play a vital role in indexing scientific data for bibliometric analysis [40]. The study period is 10 years (2013–2022) [41]. The year 2023 is eliminated as the year is in progress. By limiting subject area (Business, Management and Accounting; and Economics, Econometrics and Finance) [32], document type (Article), publication stage (Final), sources (Journal) [42], and language (English) [42], we got 1256 documents from Scopus database. Similarly, 209 articles were extracted from WOS by limiting subject area (Management, Economics and Economic Theory), period (2013–2022), document type (Article), WOS categories (Business, Management, Economics, Operations research management science, Business finance, Industrial Relations labour, Development studies, and ethics), language (English), and social science citation index. The final dataset comprises 1300 articles after eliminating 165 duplicate documents from the respective databases. This study has concentrated on articles only to include only relevant and impact-worthy studies pertaining to business and allied domains [43].

Performance analysis and science mapping in bibliometrics are complex. Often relying on one software does not follow a complete recommended workflow of bibliometrics [44]. We have used VOSviewer [45], R-studio [46] and MS Excel for performance analysis and science mapping [47]. Performance analysis includes the distribution of publications and identification of productive and influential parameters like; authors, countries, funding agencies, affiliation, sources, and commonly occurred keywords [48]. Science mapping covers social and intellectual structure [49], out of which we concentrate only on intellectual structure. The systematic process followed is summarised below-

## 4 Data analysis

Key bibliometric data on AI obtained from Scopus and WOS database. The data were analysed to estimate the performance and to visualise the intellectual structure through co-occurrence analysis of keywords. A comparison was made over each criterion of analysis across both the database.

Table 2 extracts vital information on the evolution and patterns of published documents (see Table 1). We compare both databases to identify the leading and most impactful database in the said domain. In the “Average citations per documents”, “Co-Authors per Documents”, and “International co-authorships %” criteria WOS takes the lead. But the Scopus database leads the list in the rest of the criteria.

**Table 1:** Parameters of data analysis.

Broad Analysis	Sub-Analysis	Data	Parameters	Remarks
General	Comparison between 2 databases on extracted data	Number of publications, sources, keywords, authors and citation	Actual numbers	[50]
Performance	Research Trends and productive year	Document & Citation	Actual/proportionate	[51]
	Productive & prominent country, affiliation, funding agency, source and author	Document & Citation	Actual/proportionate	[51]
Science Mapping	Co-occurrence	Authors keywords	TLS/Occurrence	[47,52,53]

**Table 2:** Comparison of reviewed publications.

Particulars	Scopus	Web of Science	Comparison
Timespan	2013–2022	2013–2022	Same
Sources (Journals, Books, etc.)	527	121	Scopus
Articles	1256	209	Scopus
Annual Growth Rate %	63.7	19.62	Scopus
Document Average Age	2.51	2.13	Scopus
Average citations per document	22.68	34.90	Web of Science
References	71,332	13,318	Scopus
Keywords Plus	2925	660	Scopus
Authors	3100	589	Scopus
Single-authored documents	291	20	Scopus
Co-Authors per Documents	2.89	3.1	Web of Science
International co-authorships %	30.9	52.63	Web of Science

In the last 10 years, 1256 documents were indexed in Scopus whereas 209 were in WOS. The annual growth rate of Scopus and WOS is 63.7 % and 19.62 %, respectively. The sources for Scopus are 527, while for WOS is 121.2925 keywords are recorded in Scopus while 660 keywords are in WOS. The average document age of Scopus is 2.51, whereas WOS is 2.13.

The average citation per document is 22.68 and 34.56, respectively, for Scopus and WOS. 291 ‘Single authored documents’ are identified from Scopus whereas 20 are from WOS. 3100 authors have published their research outcomes in Scopus-indexed journals, whereas 589 researchers have published in WOS indexed journals. It is clear that the authors have a high preference for Scopus indexed journals, but the documents of WOS got the maximum number of citations per document in the said domain.

**Fig. 1:**
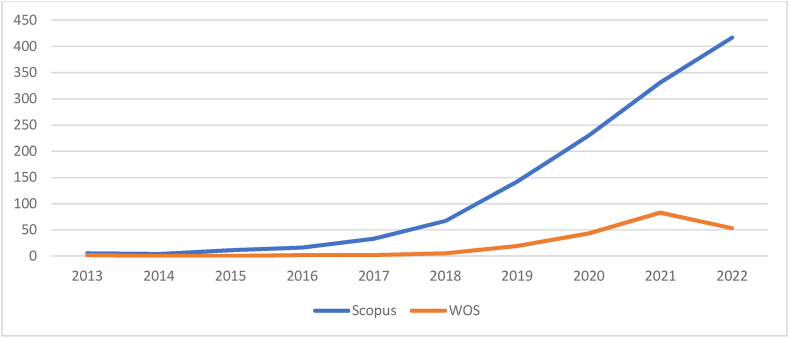
Year-wise analysis of documents. Legends: WOS- Web of Science.

**Fig. 2:**
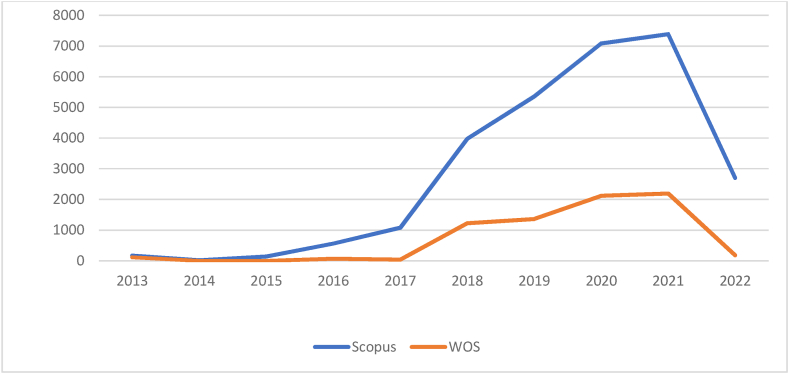
Year-wise analysis of citation. Legends: WOS- Web of Science.

Table 3 and corresponding Fig. 1, Fig. 2 summarise the chronological progress in AI research. In the Scopus database, 1256 documents are indexed. Whereas in WOS, 209 documents are indexed. In WOS, no documents were indexed between 2013 and 2014. In the Scopus database, 16.32 % of documents have no citation, but in WOS, it is only 4.78 %. The average citations per document is 22.68 and 34.90 for Scopus and WOS, respectively. It is clear that Scopus is a popular database with a wide range of coverage of documents, but the impact of WOS documents is more as it has the highest average citations and less percentage of non-cited documents.

**Table 3:** Year-wise analysis of documents.

SN	Year	Scopus					Web of Science				
		NOD	TC	NCD	CD	Average	NOD	TC	NCD	CD	Average
1	2013	5	170	1	4	34.00	2	115	0	2	57.50
2	2014	4	21	1	3	5.25	0	0	0	0	–
3	2015	11	140	4	7	12.73	0	0	0	0	–
4	2016	16	559	7	9	34.94	2	63	0	2	31.50
5	2017	33	1078	11	22	32.67	2	36	0	2	18.00
6	2018	67	3975	11	56	59.33	5	1224	0	5	244.80
7	2019	142	5362	21	121	37.76	19	1366	0	19	71.89
8	2020	230	7088	14	216	30.82	43	2116	1	42	49.21
9	2021	331	7393	35	296	22.34	83	2193	1	82	26.42
10	2022	417	2703	100	317	6.48	53	183	8	45	3.45
**Total**		**1256**	**28,489**	**205**	**1051**		**209**	**7296**	**10**	**199**	
**Average**		**125.6**	**22.68**	**10.5**	**105.1**		**20.9**	**34.90**	**1**	**19.9**	

Legends: NOD- No of Documents, TC- Total Citation, NCD- Total Non-Cited Documents, CD- Total Cited Documents.

(Source: Compiled and Computed from Scopus and Web of Science Database over a period from January 1, 2013 to December 31, 2022)

(Source: Compiled and Computed from Scopus and Web of Science Database over a period from January 1, 2013 to December 31, 2022)

A detailed analysis has been made, segregating the study period into two quinquennials. During the first quinquennial (2013–2017), the research on AI was not popular, as only 69 and 6 documents were indexed in Scopus and WOS, respectively. In the second quinquennium, the momentum gained in AI research. During this period, more than 17 and 33 times of documents were indexed, respectively, in Scopus and WOS. A similar trend was also observed in citations for both the database. The study argues the increasing importance of AI for decision-making in business management.

**Fig. 3:**
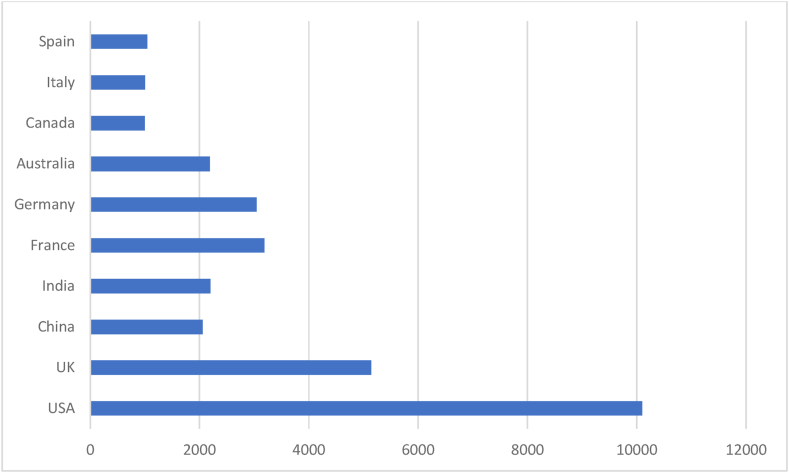
Prominent countries citations (scopus).

Source: Compiled and Computed from Scopus Database over a period from January 1, 2013 to December 31, 2022

**Fig. 4:**
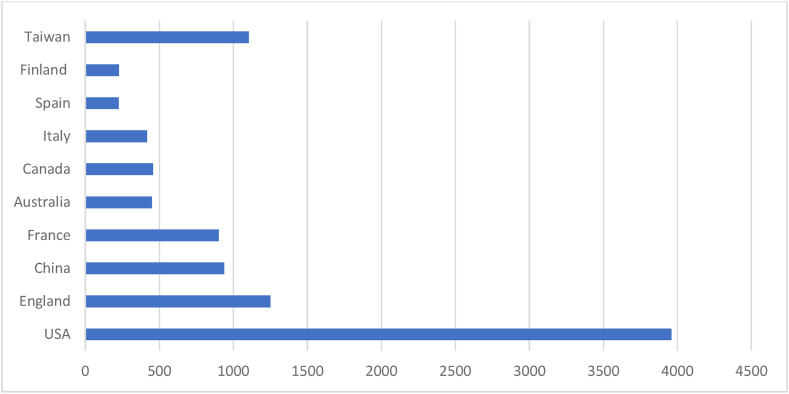
Prominent countries citations (web of science).

Source: Compiled and Computed from Web of Science Database over a period from January 1, 2013 to December 31, 2022

Table 4 and corresponding Fig. 3 & Fig. 4 summarise the most productive countries in AI research. The table enlightens on performance indicators like documents, citations, non-cited documents, and cited documents, whereas the graph expresses the citations. The USA, UK and China are the top three contributing countries. Jointly these 3 counties have contributed more than 50 % of the documents the prominent countries contributed. Regarding citations, the USA, UK and France hold the top three positions in the Scopus database. But the USA, UK and Taiwan hold the top three positions in the WOS database. The result suggests USA and UK are the leading countries in AI research. The findings of this analysis argue advanced research on AI is taken care of by most developed economies.

**Table 4:** Prominent countries.

SL No	Scopus						Web of Science					
	Country	NOD	TC	NCD	CD	Average	Country	NOD	TC	NCD	CD	Average
1	USA	269	10,095	23	246	37.53	USA	66	3960	2	64	60.00
2	UK	152	5146	12	140	33.86	England	42	1250	0	44	29.76
3	China	128	2053	27	101	16.04	China	38	937	4	34	24.66
4	India	128	2196	26	102	17.16	France	24	902	1	23	37.58
5	France	90	3186	7	93	35.40	Australia	18	450	2	16	25.00
6	Germany	89	3045	6	83	34.21	Canada	16	457	1	15	28.56
7	Australia	69	2186	4	65	31.68	Italy	14	418	0	14	29.86
8	Canada	49	999	5	44	20.39	Spain	11	224	0	11	20.36
9	Italy	44	1000	5	39	22.73	Finland	10	227	0	10	22.70
10	Spain	41	1042	5	36	25.41	Taiwan	10	1104	0	10	110.40

Legends: NOD- No of Documents, TC- Total Citation, NCD- Total Non-Cited Documents, CD- Total Cited Documents.

*Here we have considered UK and England as same.

**Fig. 5:**
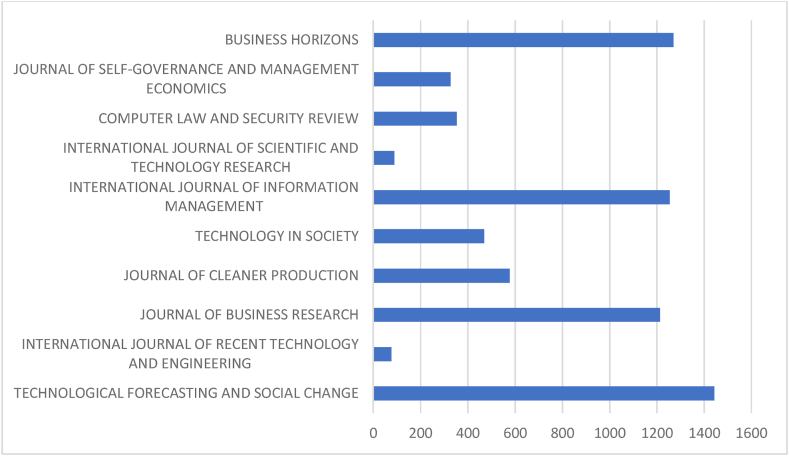
Citations of prominent sources (scopus).

Source: Compiled and Computed from Scopus Database over a period from January 1, 2013 to December 31, 2022

**Fig. 6:**
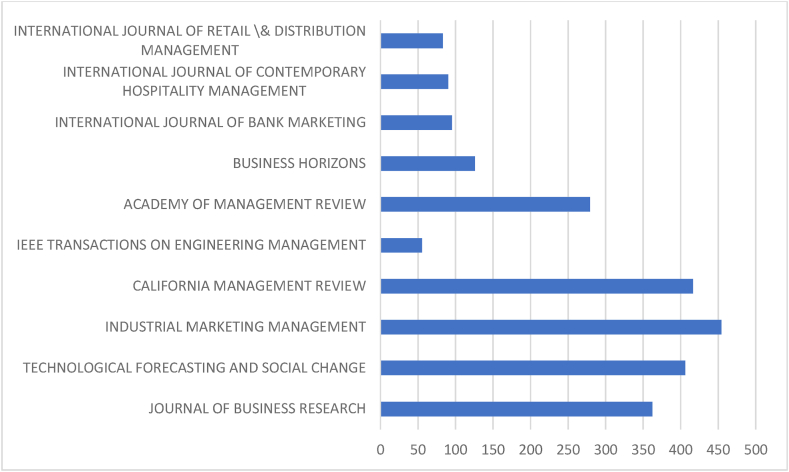
Citations of prominent sources (web of science).

Source: Compiled and Computed from Web of Science Database over a period from January 1, 2013 to December 31, 2022

Table 5 and corresponding Fig. 5 & Fig. 6 discuss prominent sources. Prioritising journal publications, the performance analysis of journals have been carried out to identify the most productive and influential journals from both databases. The comparison criterion is ‘Proportion’ and ‘Total’. “Technological Forecasting and Social Change”, “International Journal of Recent Technology and Engineering” and “Journal of Business Research” are prominent sources in the Scopus database with 1443, 76 and 1213 citations from 32, 27 and 25 documents, respectively. Similarly, “Journal of Business Research”, “Technological Forecasting and Social Change” and “Industrial Marketing Management”, with 362, 406 and 454 citations from 13, 13 and 8 documents, lead the list in the WOS database. “Technological Forecasting and Social Change”, “Journal of Business Research”, and “Business Horizons” are the prominent sources of both databases. These three journals have prominent positions in the Scopus and WOS database with inter-changeable positions. WOS-indexed sources lead the list on proportion, whereas Scopus-indexed sources in the latter. The result indicates greater outreach of Scopus and higher popularity and impact of WOS sources.

**Table 5:** Prominent sources.

SL No	Scopus						Web Of Science					
	Sources	NOD	Tc	H	G	M	Sources	NOD	Tc	H	G	M
1	Technological Forecasting and Social Change	32	1443	17	32	2.13	Journal of Business Research	13	362	10	13	3.33
2	International Journal of Recent Technology and Engineering	27	76	6	8	1	Technological Forecasting and Social Change	13	406	8	13	2
3	Journal Of Business Research	25	1213	16	25	4	Industrial Marketing Management	8	454	6	8	0.55
4	Journal Of Cleaner Production	24	577	13	24	1.86	California Management Review	4	416	4	4	0.8
5	Technology In Society	19	469	12	19	2	Ieee Transactions on Engineering Management	5	55	4	5	1.33
6	International Journal of Information Management	17	1254	12	17	2.4	Academy Of Management Review	3	279	3	3	1
7	International Journal of Scientific and Technology Research	16	89	4	9	0.67	Business Horizons	3	126	3	3	0.6
8	Computer Law and Security Review	15	353	8	15	0.89	International Journal of Bank Marketing	3	95	3	3	0.5
9	Journal Of Self-Governance and Management Economics	14	327	11	14	1.83	International Journal of Contemporary Hospitality Management	3	90	3	3	1
10	Business Horizons	13	1269	12	13	2	International Journal of Retail & Distribution Management	3	83	3	3	0.6
**Total**		**202**	**7070**				**Total**	**58**	**2366**			
**Proportion to Total**		**16.08**	**24.82**				**Proportion To Total**	**27.75**	**32.43**			

Legends: NOD- No of Documents, TC- Total Citation, h-h index, g-g index, m-m index.

**Fig. 7:**
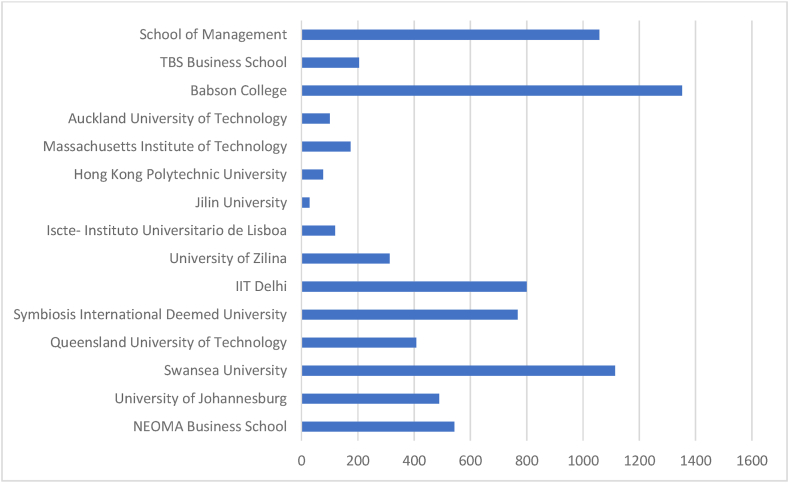
Citations of prominent Affiliation's (scopus).

Source: Compiled and Computed from Scopus Database over a period from January 1, 2013 to December 31, 2022

**Fig. 8:**
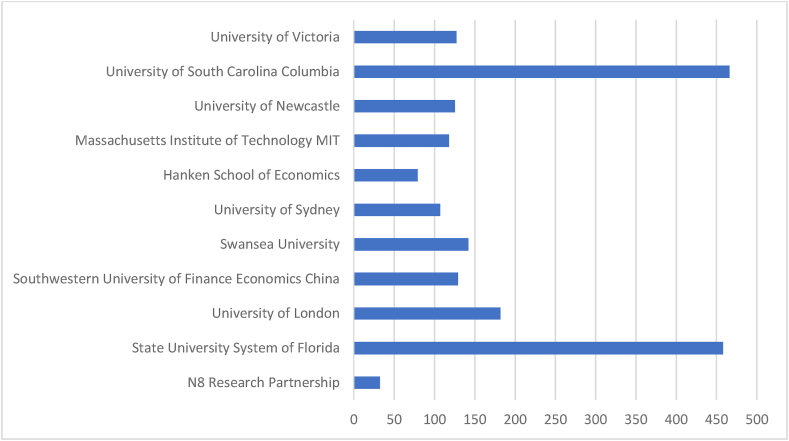
Citations of prominent Affiliation's (web of science).

Source: Compiled and Computed from Web of Science Database over a period from January 1, 2013 to December 31, 2022

Table 6 and corresponding Fig. 7 & Fig. 8 summarise the highly productive affiliations. Prominent affiliating institutions are identified from Scopus and WOS databases considering the number of documents produced and citations. Affiliations from Scopus-indexed documents are advantageous in total documents, citations, and CD. But institutions identified from WOS documents have prominence in terms of ‘Proportionate’ values.

**Table 6:** Prominent affiliations.

SL No	Scopus						Web of Science					
	Name of The Organisation	NOD	TC	NCD	CD	Average	Name of The Organisation	NOD	TC	NCD	CD	Average
1	NEOMA Business School	13	542	0	13	41.69	N8 Research Partnership	9	32	0	9	3.56
2	University of Johannesburg	11	489	1	10	44.45	State University System of Florida	7	458	0	7	65.43
3	Swansea University	11	1114	0	11	101.27	University of London	7	182	0	7	26.00
4	Queensland University of Technology	11	407	2	9	37.00	Southwestern University of Finance Economics China	5	129	0	5	25.80
5	Symbiosis International Deemed University	11	768	0	11	69.82	Swansea University	5	142	0	5	28.40
6	IIT Delhi	10	799	0	10	79.90	University of Sydney	5	107	1	4	21.40
7	University of Zilina	9	313	0	9	34.78	Hanken School of Economics	4	79	0	4	19.75
8	Iscte- Instituto Universitario de Lisboa	9	119	2	7	13.22	Massachusetts Institute of Technology MIT	4	118	0	4	29.50
9	Jilin University	8	28	1	7	3.50	University of Newcastle	4	125	0	4	31.25
10	Hong Kong Polytechnic University	8	77	1	7	9.63	University of South Carolina Columbia	4	466	0	4	116.50
11	Massachusetts Institute of Technology	8	173	2	6	21.63	University of Victoria	4	127	0	4	31.75
12	Auckland University of Technology	8	99	0	8	12.38						
13	Babson College	8	1352	0	8	169.00						
14	TBS Business School	8	204	0	8	25.50						
15	School of Management	8	1058	0	8	132.25						
	**Total (Overall)**	**141**	**7542**	**9**	**132**		**Total (Overall)**	**58**	**1965**	**1**	**57**	
	**Proportion to grand total**	**11.2**	**26.47**	**4.39**	**12.6**		**Proportion to grand total**	**27.8**	**26.93**	**10**	**29**	

Legends: NOD- No of Documents, TC- Total Citation, NCD- Total Non-Cited Documents, CD- Total Cited Documents.

“NEOMA Business School” has the highest 13 documents in the Scopus database, whereas “N8 Research Partnership” has the highest 9 documents from the WOS database. From the citations point of view, “Babson school” has the highest 1352 TC from 8 documents in the Scopus database. But in the WOS database, “University of South Carolina Columbia” has the highest 466 citations from 4 documents. We observed that WOS has a higher percentage of NCD. The minimum average citation per document of both databases is nearly the same, i.e., approximately 4. But the highest average citation per document is 169 and 117, respectively, for Scopus and WOS.

**Fig. 9:**
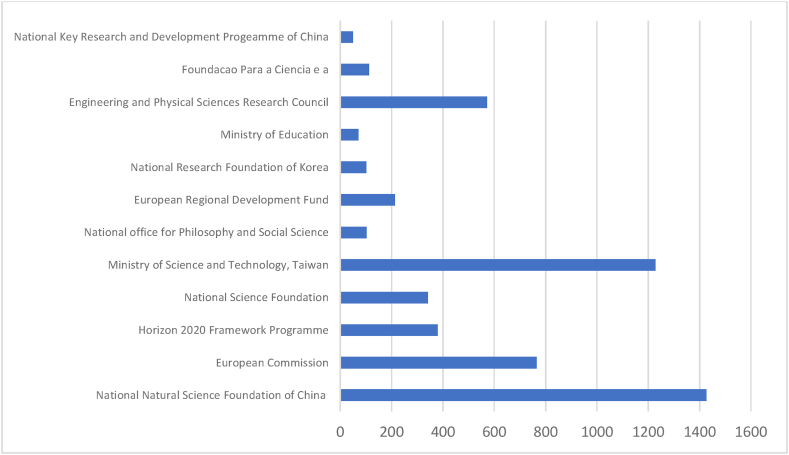
Citations of prominent funding agencies (scopus).

Source: Compiled and Computed from Scopus Database over a period from January 1, 2013 to December 31, 2022

**Fig. 10:**
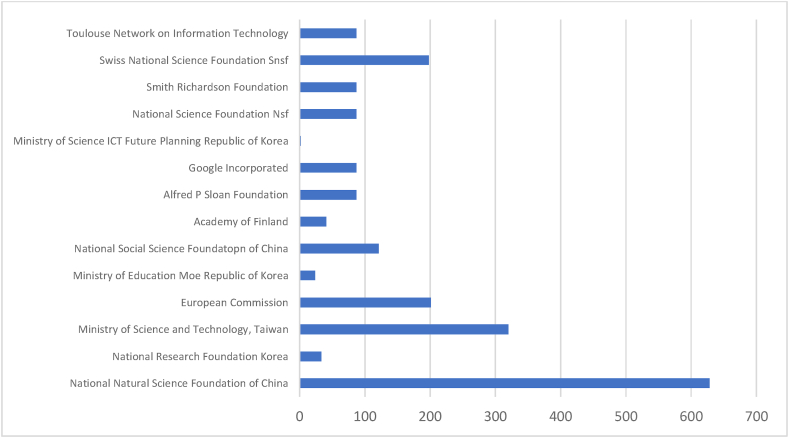
Citations of prominent funding agencies (web of science).

Source: Compiled and Computed from Web of Science Database over a period from January 1, 2013 to December 31, 2022

Table 7 and corresponding Fig. 9 & Fig. 10 summarise the performance of the prominent funding agencies referring to documents produced and citations received. The range of documents published by prominent funding agencies is 5–50 in Scopus, whereas 2 to 14 in WOS. The top three funding agencies are accountable for at least 50 % of the documents produced by all prominent agencies. Among Scopus-indexed documents, The National Natural Science Foundation of China (50), the European Commission (15), and the Horizon 2020 Framework Programme (15) are the three most prominent funding agencies. These three agencies have 2567 cumulative citations. The National Natural Science Foundation of China (14), the National Research Foundation Korea (6), and the Ministry of Science and Technology, Taiwan (5) are the three most prominent funding agencies identified from WOS. The cumulative citation of these three agencies is 981. All 50 documents indexed in WOS have minimum citations of 1. Out of 156 documents indexed in Scopus, 12 documents have 0 citations. The result confirms that WOS-indexed documents have more consistent citations than Scopus. The Scopus database leads the list in total publications and citations. WOS leads the list in each parameter on the proportionate criterion of analysis.

**Table 7:** Prominent funding agencies.

SL NO	Scopus						Web of Science					
	Funding Sponsor	NOD	TC	NCD	CD	Average	Funding Sponsor	NOD	TC	NCD	CD	Average
1	National Natural Science Foundation of China	50	1427	7	43	28.54	National Natural Science Foundation of China	14	628	0	14	44.86
2	European Commission	15	764	0	15	50.93	National Research Foundation Korea	6	33	0	6	5.50
3	Horizon 2020 Framework Programme	15	380	0	15	25.33	Ministry of Science and Technology, Taiwan	5	320	0	5	64.00
4	National Science Foundation	15	341	1	14	22.73	European Commission	3	201	0	3	67.00
5	Ministry of Science and Technology, Taiwan	11	1229	2	9	111.73	Ministry of Education Moe Republic of Korea	3	24	0	3	8.00
6	National office for Philosophy and Social Science	11	103	2	9	9.36	National Social Science Foundation of China	3	121	0	3	40.33
7	European Regional Development Fund	9	212	0	9	23.56	Academy of Finland	2	41	0	2	20.50
8	National Research Foundation of Korea	9	100	0	9	11.11	Alfred P Sloan Foundation	2	87	0	2	43.50
9	Ministry of Education	6	70	0	6	11.67	Google Incorporated	2	87	0	2	43.50
10	Engineering and Physical Sciences Research Council	5	571	0	5	114.20	Ministry of Science ICT Future Planning Republic of Korea	2	2	0	2	1.00
11	Foundacao Para a Ciencia e a	5	112	0	5	22.40	National Science Foundation Nsf	2	87	0	2	43.50
12	National Key Research and Development Progeamme of China	5	50	0	5	10.00	Smith Richardson Foundation	2	87	0	2	43.50
13							Swiss National Science Foundation Snsf	2	198	0	2	99.00
14							Toulouse Network on Information Technology	2	87	0	2	43.50
	**Total**	**156**	**5359**	**12**	**144**		**Total**	**50**	**2003**	**0**	**50**	

Legends: NOD- No of Documents, TC- Total Citation, NCD- Total Non-Cited Documents, CD- Total Cited Documents.

Table 8 summarises the prominent authors from Scopus and WOS databases. The parameters of analysis are document produced and citations. The 10 productive authors from Scopus are accountable for little less than 5 % of total documents. Out of 61 documents of the most productive authors, little less than 10 % have funding support, whereas 88.52 % have a minimum of 1 citation. The range of authors' productivity (h-index), impact (g-index), and average citation per publication (m-index) of the ten most productive authors are ‘1 to 8’, ‘1 to 8’ and 0.5 to 2.667, respectively. The 10 productive authors from WOS are accountable for little less than 12 % of total documents. Out of 25 documents, little less than 36 % have funding support, whereas all documents have a minimum of 1 citation. The range of authors' productivity, impact and average citation per publication is ‘1 to 3’, ‘2 to 3’ and 0.33 to 0.667, respectively. On the productivity parameter, impact and average citation per publication, Scopus is in an advantageous position. But in cited documents and funded documents, WOS leads the list.

**Table 8:** Prominent author analysis.

Scopus										
Authors	T Doc	F Doc	NF Doc	CD	NCD	TC	AC	H	g	M
Dwivedi, Y.K.	8	4	4	8	0	1088	136.00	8	8	2.667
Haenlein, M.	7	0	7	7	0	792	113.14	6	7	1
Dias, A.	6	0	6	4	2	6	1.00	1	1	0.5
Fosso Wamba, S.	6	0	6	6	0	179	29.83	5	6	1.25
Goncalves, R.	6	0	6	4	2	6	1.00	2	2	1
Kaplan, A	6	0	6	6	0	717	119.50	5	6	0.833
Kietzmann, J.	6	0	6	6	0	281	46.83	5	6	1
Pereira, L.	6	0	6	4	2	6	1.00	2	2	1
Chatterjee, S.	5	0	5	5	0	168	33.60	4	5	1
Desouza, K.C	5	2	3	4	1	187	37.40	4	5	1
**Total**	**61**	**6**	**55**	**54**	**7**	**3430**	**51.93**			
**Web of Science**										
**Authors**	**T Doc**	**F Doc**	**NF Doc**	**CD**	**NCD**	**TC**	**AC**	**H**	**G**	**M**
Kietzmann, J	3	0	3	3	0	114	38.00	2	3	0.4
Gans, Joshua S.S.	3	0	3	3	0	143	47.67	2	3	0.4
Mikalef, Patrick	3	0	3	3	0	166	55.33	2	2	0.667
Malik, Ashish	3	1	2	3	0	33	11.00	2	2	0.667
Huang, Minghui	3	2	1	3	0	1008	338.00	3	3	0.5
Luo, Xueming	2	1	1	2	0	254	127.00	2	2	0.4
Lu, Chia-Hui	2	2	0	2	0	9	4.50	1	2	0.33
Prentice, C	2	0	2	2	0	136	68.00	2	2	0.5
Fang, Zheng	2	1	1	2	0	254	127.00	2	2	0.4
Krakowski, Sebastian	2	2	0	2	0	201	100.50	2	2	0.667
**Total**	**25**	**9**	**16**	**25**	**0**	**2318**	**91.70**			

Legends: T Doc- Total no of Documents, F Doc- Funded Documents, NF Doc- Non-Funded Documents, TC- Total Citation, NCD- Total Non-Cited Documents, CD- Total Cited Documents, AC- Average Citation, h-h index, g-g index, m-m index.

From the list of 20 prominent authors ‘Kietzmann, J’ is the only common author in both databases. In Scopus ‘Dwivedi, Y.K.’ is the most productive author with 8 documents, followed by Haenlein, M. with 7 documents. Dwivedi, Y.K have a total of 1088 citations, out of which [18] has the highest 626 citations. In WOS, five authors have produced 3 documents each. Of these five authors, ‘Huang Minghui’ have the highest citations (1008). Of the three documents, [54] has the highest (731) citations. ‘Dwivedi, Y.K.’ and ‘Huang, Minghui’ are the most productive authors from Scopus and WOS databases in AI.

## 5 Co-occurrence analysis

Table 9 summarises the most frequently used keywords. The Authors' keywords analysis aims to identify the most prominent keywords. Co-occurrence analysis, followed by keyword analysis, aims to map issues discussed so far and issues that have significant importance in the present and future. The most frequently used 15 keywords are identified by analysing the documents. Out of these 15 keywords, 6 are commonly found in both the index and the database created from the mix of both indices. These keywords are Artificial Intelligence, Machine Learning, Big Data, AI, Automation and Artificial Intelligence (AI). Of the 15 keywords, Decision Making, Innovation, Ethics, Knowledge Management, Entrepreneurship, Sustainability and Industry 4.0 are unrelated to technical aspects. These keywords indicate modern businesses' dependency on AI's application in the decision-making and sustainability of the business. Across three databases, Artificial Intelligence, Machine Learning and Big Data are the most frequently used keywords. In Scopus, the occurrence of Artificial Intelligence, Machine Learning and Big Data is 875, 141 and 70, whereas in WOS, it is 139,18 and 9, respectively.

**Table 9:** Co-occurrence with authors' keywords.

SL No	Scopus			Web of Science			Scopus + Web of Science		
	Keywords	TLS	OCC	Keywords	TLS	OCC	Keywords	TLS	OCC
1	Artificial Intelligence	936	875	Artificial Intelligence	139	139	Artificial Intelligence	563	799
2	Machine learning	261	141	Machine learning	34	18	Machine Learning	186	127
3	Big Data	156	70	Big Data	22	9	Big Data	109	56
4	Decision Making	114	46	Artificial Intelligence (AI)	20	15	AI	77	56
5	Automation	111	44	AI	16	9	Automation	72	36
6	AI	96	57	Entrepreneurship	15	6	Ethics	58	32
7	Innovation	90	36	Decision Making	14	5	Artificial Intelligence (AI)	52	63
8	Artificial Intelligence (AI)	88	59	Automation	13	5	Industry 4.0	44	25
9	Internet of Things	85	38	Knowladge Management	11	5	Sustainability	44	24
10	Ethics	82	34	Technological Innovation	11	4	Internet of Things	43	29

Legends: TLS- Total Link Strength, OCC- Occurrence.

**Fig. 11:**
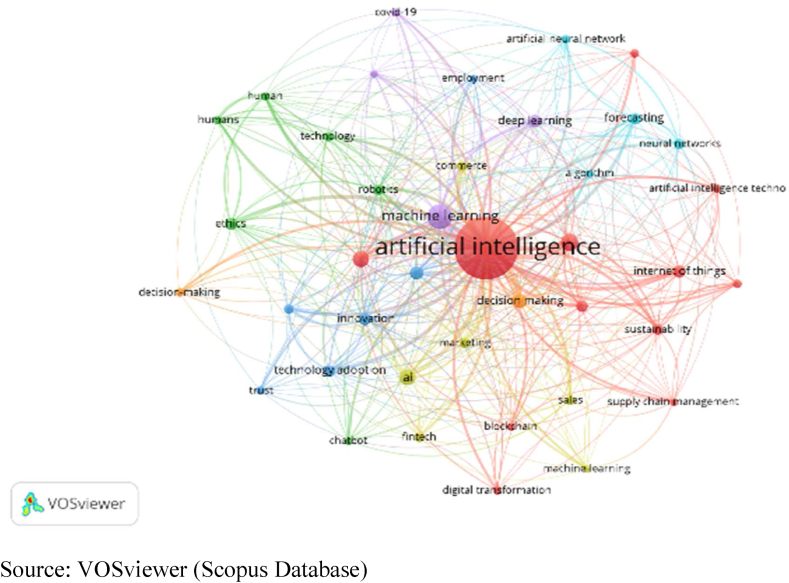
Cluster analysis of Co-Occurrence with Authors Keywords (Scopus).

Source: VOSviewer (Scopus Database)

**Fig. 12:**
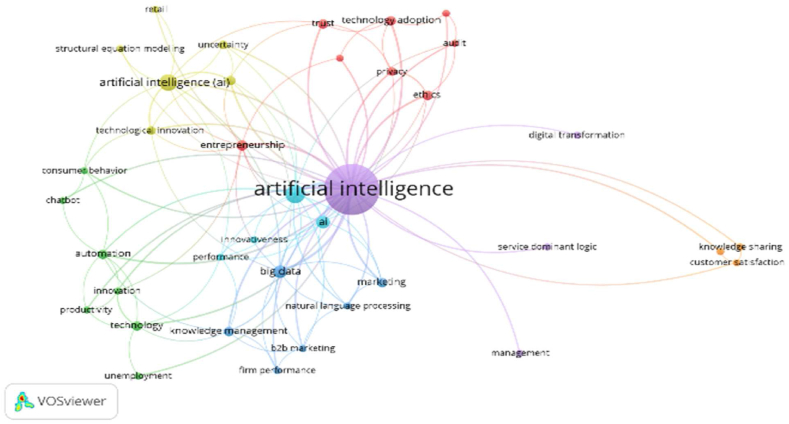
Cluster analysis of Co-Occurrence with Authors Keywords (Web of Science).

Source: VOSviewer (Web of Science Database)

**Fig. 13:**
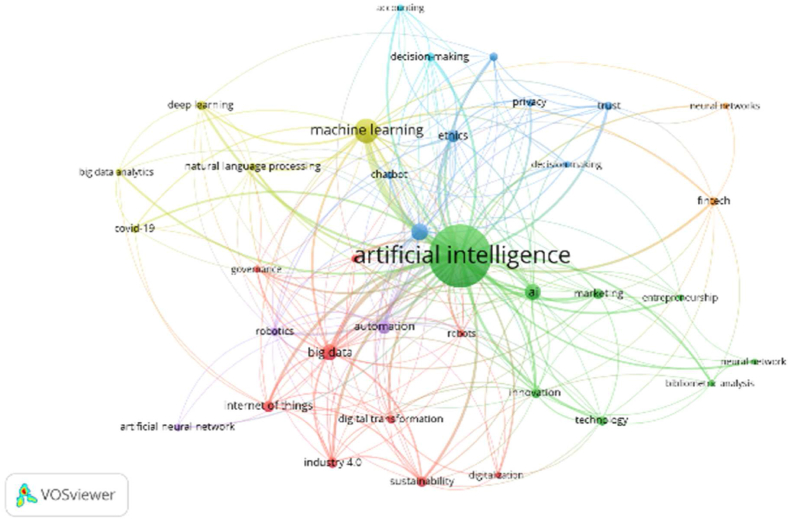
Cluster analysis of Co-Occurrence with Authors Keywords (Scopus + Web of Science).

Source: VOSviewer (Scopus + Web of Science Database)

Table 10 and corresponding Fig. 11, Fig. 12 & Fig. 13 explain the Co-Occurrence of the Authors’ Keywords. Forty keywords are selected from Scopus documents in the domain of AI and its surrounding terms. The keywords are classified into 7 clusters. Cluster-1 (green dots) contains 12 keywords focused on integrating AI and business decision-making. Out of these keywords, Human Resource Management, Supply Chain Management, Sustainability and Sustainable Development are the non-AI keyword. The occurrence and TLS are 1202 and 1609, respectively, for these 12 keywords. Similarly, cluster-2 attempts on ethical aspects, cluster-3 on employment, cluster-4 on technology adoption for business development, cluster-5 on Covid −19 and cluster-6 & 7 on forecasting and decision-making aspects of management in the presence of AI.

**Table 10:** Cluster analysis of Co-Occurrence with Authors Keywords.

Scopus								
CN	Item (Author Keywords)	NOD	L	TLS	OCC	H & L TLS	Name of The Cluster	Colour of Cluster
1	Artificial Intelligence, Artificial Intelligence (AI), Artificial Intelligence Technologies, Big Data, Blockchain, Digital Transformation, Human Resource Management, Industry 4.0, Internet Of Things, Supply Chain Management, Sustainability, Sustainable Development	12	263	1609	1202	Artificial Intelligence and Human Resource Management	Artificial Intelligence	Green
2	Chatbot, Ethics, Human, Humans, Robotics, Technology	6	121	374	131	Ethics and Chatbot	Ethics	Yellow
3	Automation, Employment, Innovation, Technological Development, Technology Adoption, Trust	6	120	399	162	Automation and Trust	Automation	Blue
4	AI, Commerce, Fintech, Machine-Learning, Marketing, Sales	6	119	349	162	AI and Fintech	AI	Red
5	Covid-19, Deep Learning, Machine Learning, Natural Language Processing	4	91	401	208	Machine Learning and Covid-19	Machine Learning	Dark Blue
6	Algorithm, Artificial Neural Network, Forecasting, Neural Networks	4	70	201	91	Forecasting and Artificial Neural Network	Forecasting	Light Blue
7	Decision Making, Decision-Making	2	44	145	62	Decision Making and Decision-Making	Decision Making	Orange
**Web of Science**								
1	Audit, Audit Quality, Digital Entrepreneurship, Entrepreneurship, Ethics, Privacy, Technology Adoption, Trust	8	39	66	34	Entrepreneurship and Audit Quality	Entrepreneurship	Red
2	Automation, Chatbot, Consumer Behaviour, Innovation, Productivity, Technology, Unemployment	7	34	54	25	Automation and Unemployment	Automation	Green
3	B2B Marketing, Big Data, Firm Performance, Knowledge Management, Marketing, Natural Language Processing	6	40	65	28	Big Data and Firm Performance	Big Data	Light Blue
4	Artificial Intelligence (AI), Decision Making, Retail, Structural Equation Modeling, Technological Innovation, Uncertainty	6	33	56	34	Artificial Intelligence (AI) and Structural Equation Modeling	Artificial Intelligence (AI)	Yellow
5	Artificial Intelligence, Digital Transformation, Management, Service Dominant Logic	4	40	147	148	Artificial Intelligence and Digital Transformation	Artificial Intelligence	Purple
6	AI, Innovativeness, Machine Learning, Performance	4	35	61	33	Machine Learning and Innovativeness	Machine Learning	Dark Blue
7	Customer Satisfaction, Hotels, Knowledge Sharing	3	9	13	9	Hotels and Customer Satisfaction	Hotels	Orange
**Scopus + Web of Science**								
1	Big Data, Blockchain, Digital Transformation, Digitalization, Governance, Industry 4.0, Internet of Things, Robots, Sustainability	9	124	360	196	Big Data and Digitalization	Big Data	Red
2	AI, Artificial Intelligence, Bibliometric Analysis, Entrepreneurship, Innovation, Marketing, Neural Network, Technology	8	119	793	859	Artificial Intelligence and Neural Network	Artificial Intelligence	Green
3	Artificial Intelligence (AI), Chatbot, Decision Making, Ethics, Privacy, Technology Adoption, Trust	7	96	241	165	Ethics and Decision Making	Ethics	Dark Blue
4	Big Data Analytics, Covid-19, Deep Learning, Machine Learning, Natural Language Processing	5	59	280	183	Machine Learning and Big Data Analytics	Machine Learning	Yellow
5	Artificial Neural Network, Automation, Robotics	3	41	118	64	Automation and Artificial Neural Network	Automation	Purple
6	Accounting, Decision-Making	2	18	48	27	Decision-Making and Accounting	Decision-Making	
7	Fintech, Neural Networks	2	13	30	26	Fintech, Neural Networks	Fintech	Orange

Legends: NOI–No of Documents, L- Links, TLS- Total Link Strength, OCC- Occurrence, H&L TLS- Highest and Lowest Total Link Strength.

With 38 authors' keywords, the co-occurrence analysis has been performed. The keywords from WOS sub-grouped into 7 clusters. Cluster-1 (red dots) has 8 keywords focused on AI's utility in auditing, prioritising ethics and trust. The keywords of this cluster are Audit, Audit Quality, Digital Entrepreneurship, Entrepreneurship, Ethics, Privacy, Technology Adoption and Trust. The occurrence and TLS are 34 and 66, respectively. Similarly, rest six clusters enlighten the diversified use of AI in solving different problems in business management. Cluster-2 discusses the impact of AI on productivity and employment, Cluster-3 on marketing aspects and knowledge management, Cluster-4 on decision-making in retail, Cluster-5 on service management, Cluster-6 on performance and Cluster-7 on customer satisfaction and knowledge sharing.

The combination of keywords from Scopus and WOS produces 36 keywords. Seven clusters were formed from these 36 keywords. The keywords are influenced by more technical terms. The result of cluster analysis is more similar to the results of the Scopus database. Cluster-1 (nine keywords) has two non-AI keywords. The cluster integrates AI for good governance and sustainability of business. Similarly, Cluster-2 integrate AI in entrepreneurship, innovation and marketing, Cluster-3 on decision-making, Cluster-4 on Covid-19, and Cluster-6 and 7 on accounting, decision making and fintech.

## 6 Authors productivity (Lotka's law)

**Fig. 14:**
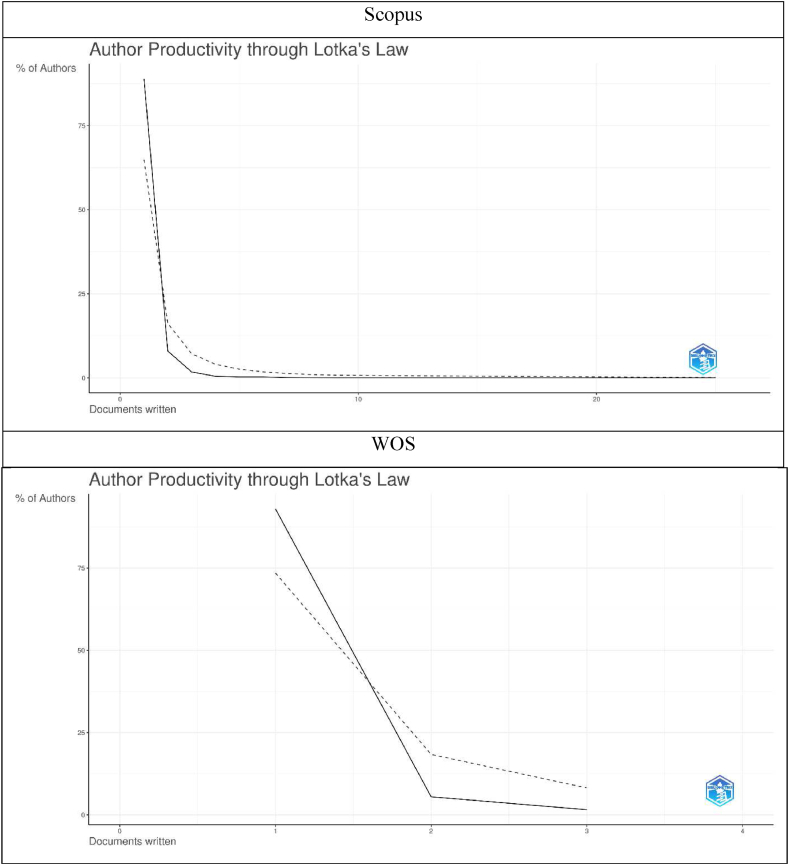
Lotka's law.

Sources: Compiled and computed data

Fig. 14 presents a comparative analysis of authors productivity through Lotka's Law. As evident, the majority of authors are found to produce only one document each. For Scopus database, 2745 authors (89 %) have produced only 1 document in the said field. While for WOS, 119 authors (93 %) have produced the same. It is to be noted that only a handful of authors have contributed scholarly works in the said field in both databases. This attracts scholarly intervention for producing more number of impactful research work in the said field.

## 7 Findings

The study seeks to achieve its objectives by performing a bibliometric analysis. The primary focus of the study is to conduct a bibliometric workflow analysis to recognise different networks and metrics. Comprehensive performance and co-occurrence analysis are carried out to crystalise the future growth and research trends in AI. The major findings of the study are discussed below.

Scopus leads the segment in terms of research consistency while WOS has the highest research impact. *“Dwivedi, Y.K.”* is identified as the most impactful researcher for Scopus, whereas *“Huang, Minghui”* is for the latter. Developed countries are found to have higher impactful research. China is the most promising country in terms of research funding. “Machine learning”, “neural networks”, and “blockchain” are some technical trending themes in the field of AI research. About 90 % of documents have been produced by single authors in both the database.

## 8 Discussion

The number of AI publications per year is rising in both the database. The growth rate of publication and coverage of Scopus is consistent, while the publication impact of WOS is higher. The most productive years in AI research are 2022 for Scopus and 2021 for WOS. Author *“Dwivedi, Y.K.”* stands out as the most productive and impacted author for the Scopus database. In the WOS database, *“Huang, Minghui*” is the most impacted author with 1008 citations from 3 documents, of which 2 have funding support. “*Huang, Minghui*” is the most impacted author in the AI research with the highest average citation per document.

The leading countries in AI research are USA, UK and China, with the highest document production in both databases. Among prominent countries, USA and Taiwan have the highest impact, respectively, in Scopus and WOS. “National Natural Science Foundation of China” is the most productive funding agency. “Babson College”, “School of Management” and “Swansea University” are the prominent affiliating institutions for ‘Scopus’ while “The University of South Carolina Columbia” and “State University System of Florida” for WOS indexed documents.

In the Scopus database, the most productive sources are “Technological Forecasting and Social Change”, “International Journal of Recent Technology and Engineering” and “Journal of Business Research”. Whereas in the WOS, “Journal of Business Research”, “Technological Forecasting and Social Change” and “Industrial Marketing Management” are the most productive sources. Among the top 3 prominent sources “Technological Forecasting and Social Change” and “Journal of Business Research” are common in both databases. These sources have a maximum h-index than other sources, which indicates more consistency in publishing documents and gaining citations.

Keyword analysis is one of the important analyses to know the current and expected future trends. Among all keywords, “Artificial Intelligence” is the most frequently used word. “Machine learning”, “neural networks”, and “blockchain” are some trending themes in the technical field. AI research has a presence in the areas of business management. It focuses on issues like ‘Sustainability’, ‘sustainable development’, ‘accounting’, and ‘auditing’ aspects. But in turn, research on ethical aspects of AI is addressed by management research. The future trend of the research can be extended to the adverse impact of AI on employment and human resource management and the positive impact on productivity and efficiency.

## 9 Agenda for future research

**Fig. 15:**
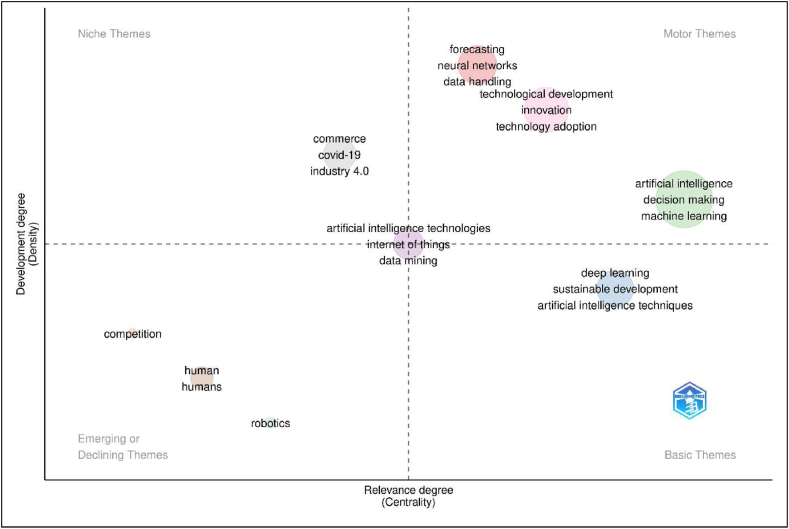
Agenda for future research.

Sources: Compiled and computed data

Fig. 15 presents a comprehensive agenda of scholarly works in the said field. The agenda can be broadly classified in to four distinctive themes such as basic themes, emerging theme, niche theme and motor theme.

The basic theme represents the blueprint of initial research works on AI. Themes such as deep learning, artificial intelligence techniques and sustainable development are classified under basic themes. The initial studies on AI represented the aforementioned themes in order to create AI software for addressing complex organisational goals, while achieving sustainable development. Deep learning assist developers to understand and comprehend the business needs and requirements of AI algorithms. Sustainable development presents the inclination of business organisations in achieving the environmental harmony using several AI techniques at their disposal.

Motor themes represent the prominent theme which are preferred for scholarly publications. Themes such as forecasting, neural network, data handling, technological development, innovation, technology adoption, artificial intelligence, decision making, and machine learning represents the most preferred themes in academia. Motor themes presented the technical framework of the AI interface using in business research. Currently AI is used in crucial business functions like innovation, leadership and decision making, and investment decisions.

Emerging themes represents the themes which are likely to be trending in the future time. Scholars across the globe are currently working on themes such as competition, human intervention and robotics. The future research trend priorities the utility of AI in securing competitive advantage across the industry. Studies are currently focusing on the human intervention and robotics on AI to achieve organisational efficiency. Future research is observed for identification of human intervention, extended use of robotics and automation as well as use of AI in strategic formulation.

Niche themes represent the dormant themes craving for scholarly attention. Themes such as commerce, Covid-19, and Industry 4.0 are classified under niche themes. Scholars are advised to study the AI implication on commerce and allied domain to achieve industry 4.0 goals. There is a latent need to comprehend the AI development and utility in managing organisational goals during Covid-19 pandemic. Research on the use of AI in handling pandemic like Covid-19 is encouraged for future researchers. While the proper use of AI for developing industry 4.0 culture in the organisation can result in an excellent finding. Although, the use of AI in business and organisation studies are more, underlying discipline such as commerce can result in interesting work.

There is a special case of themes such as artificial intelligence technologies, data mining and internet of things which have prolific impact on all the themes. The aforementioned themes are perennial in terms of scholarly works and high impact factor-based research.

## 10 Conclusion

This study examines, assesses and visualises knowledge mapping by employing performance analysis and science mapping techniques in AI. Performance analysis focuses on evaluating productivity, impact and visibility. At the same time, science mapping helps picturise the structure, trend and relations among different parameters. The concluding remarks of bibliometric analysis on AI are as follows:

The research on AI is in a growing stage. In AI research documentation, the outreach of Scopus is visible, whereas the impact of WOS is significant. USA and Taiwan are the most impactful countries, whereas USA and UK are the most productive nations. “Technological Forecasting and Social Change” and “Journal of Business Research’ are highly preferred sources. *“Dwivedi, Y.K.” and* “Huang, Minghui” are productive and impactful authors in AI research. [54] is the most impacted author in both databases.

Out of the total documents published on AI, at least 26 % of documents have any funding support. Leading software firms are extending their funding support. However, the “National Natural Science Foundation of China” is recognised as the most prominent funding agency. In AI, current research focuses on technical as well as managerial aspects. It integrates technical aspects like “Machine learning”, “neural networks”, and “blockchain” with ‘Sustainability’, ‘sustainable development’, ‘accounting’, and ‘auditing’ for managerial decision-making.

Every research has some limitations due to some unavoidable factors. The current study also has some limitations. First of all, data are collected from Scopus and WOS databases only. The results may vary if data are collected from other databases for different timeframes. In science mapping, we have analysed the co-occurrence of authors’ keywords. Other aspects of science mapping may be taken care of.

## CRediT authorship contribution statement

**Ashok Kumar Patra:** Methodology, Formal analysis, Conceptualization. **Ashyashree Praharaj:** Writing – original draft, Visualization, Methodology, Data curation, Conceptualization. **Desul Sudarshan:** Validation, Supervision, Software, Dr. **Biswajit Prasad Chhatoi:** Writing – review & editing, Validation, Supervision, Project administration, Methodology, Conceptualization.

## Declaration of competing interest

The authors declared no potential conflicts of interest with respect to the research, authorship and/or publication of this article.
